# Determinants of pregnancy success following fixed-time artificial insemination in Nelore cows under field conditions: a large-scale study

**DOI:** 10.1007/s11250-026-05319-9

**Published:** 2026-09-03

**Authors:** Arthur Ribeiro Torres, Milena Alves da Silva, Wilson Júnior Alcebíades, Adalfredo Rocha Lobo Júnior, Thiago Vasconcelos Melo, Jeanne Broch Siqueira

**Affiliations:** 1 Autonomous Veterinarian, Minas Gerais, Brazil; 2 Finom College - Campus Paracatu, Paracatu, Minas Gerais Brazil; 3 Uniatenas College, Paracatu, Minas Gerais Brazil; 4 Institute of Agricultural Sciences, Universidade Federal dos Vales do Jequitinhonha e Mucuri, Unaí – Minas Gerais, Unaí, Brazil

**Keywords:** Beef cattle, Body condition score, Progesterone device, Pregnancy rate

## Abstract

This retrospective, observational study examined the associations of female category, body condition score (BCS), timing of progesterone device removal, and repeated device use with pregnancy per fixed-time artificial insemination (FTAI) in Nelore females managed under field conditions. Records from 109 farms in Minas Gerais, Brazil, comprising 31,358 cyclic females (10,079 heifers and 21,279 multiparous cows) at their first FTAI of the breeding season, were analysed. Associations were estimated using multivariable logistic regression, which modeled all factors jointly and included farm as a random effect to account for clustering of females within farms; pairwise contrasts were adjusted for multiple comparisons, and exact p-values are reported. The overall pregnancy rate was 51.5%, with no difference between heifers (50.9%) and multiparous cows (51.8%; *p* > 0.05). In multiparous cows, BCS was positively associated with pregnancy: high-BCS cows had greater odds of pregnancy than moderate- (OR 1.90; *p* = 0.003) or low-BCS cows (OR 2.40; *p* < 0.001); no such association was observed in heifers. After multivariable adjustment and correction for clustering and multiple comparisons, the day of device removal (D8–D10) was not associated with pregnancy in any category. Pregnancy did not differ across the first three device uses; however, in multiparous cows a pre-specified contrast indicated lower odds of pregnancy at the fourth use than at earlier uses (OR 0.80; *p* = 0.028). Under these field conditions, BCS in multiparous cows was the main factor associated with pregnancy, whereas the day of device removal and device reuse up to the third time were not.

## Introduction

Brazil has one of the largest cattle herds in the world, with more than 218 million head reported in 2020 (IBGE 2023). In addition, the country has shown a consistent increase in beef exports, which grew by 11% between 2017 and 2018, reaching 1.64 million tons (ABIEC 2022). In 2019, agribusiness accounted for 21.4% of Brazil’s gross domestic product (GDP) (CEPEA 2020).

In this context, the demand for increased productivity and efficiency in cattle production has driven the adoption of technical and scientific advancements to meet both domestic and international market requirements. Improving reproductive efficiency is a key strategy to enhance herd productivity without necessarily expanding herd size, and considerable efforts have been directed toward the development and application of reproductive biotechnologies.

Artificial insemination (AI) has played a fundamental role in disseminating superior genetics with desirable zootechnical traits. However, its widespread adoption has been limited by the need for accurate estrus detection (Bisinotto and Santos 2012). This limitation has been largely overcome by the development of fixed-time artificial insemination (FTAI), which allows synchronization of ovulation and enables insemination at predetermined times without the need for estrus observation (Bó et al. 2007).

In beef cattle systems, reproductive management is also closely associated with the establishment of a defined breeding season, which contributes to greater uniformity of calves and concentration of births during periods of optimal feed availability. This practice improves herd management efficiency and production outcomes. Although body condition score, the length of progesterone exposure, and device reuse have each been investigated, evidence from large commercial datasets that evaluates these factors jointly, while accounting for the substantial variation between farms, remains limited, and reported effects of removal timing and device reuse are inconsistent across studies (Frigoni 2020). Therefore, the objective of this study was to examine the associations of female category, body condition score, timing of progesterone device removal, and repeated use of intravaginal progesterone devices (CIDR^®^) with pregnancy per FTAI in cyclic Nelore beef females managed under field conditions in Minas Gerais, Brazil.

## Materials and methods

This study was based on a retrospective, observational analysis of FTAI records from the breeding program of a private veterinary service company (Biocampo Assistência Veterinária) collected between December 2019 and April 2020.

Data were obtained from 117 farms located in the state of Minas Gerais, Brazil. During the study period, according to the National Institute of Meteorology (INMET), the temperature–humidity index (THI) remained below 76, indicating the absence of heat stress conditions that could affect reproductive performance during the FTAI protocols and pregnancy diagnosis.

A total of 31,281 Nelore females were included and categorized as heifers (*n* = 10,060) or multiparous cows (*n* = 21,221). Heifers were defined as females that had never calved, whereas multiparous cows had calved at least twice. Primiparous cows were excluded because they represented too few animals for reliable category-specific estimation in this dataset; this exclusion is acknowledged as a limitation, as primiparous females are physiologically demanding. Animals were managed under extensive grazing systems across all farms, with ad libitum access to mineral supplementation and water. Before the initiation of the hormonal protocol, all females underwent reproductive evaluation by gynecological examination, including ovarian assessment (presence of follicles and/or corpus luteum) and uterine evaluation. Only cyclic females without signs of uterine infection were enrolled in the study; females were considered cyclic when a corpus luteum and/or a functional dominant follicle was detected at gynecological examination. This selection of cyclic females is emphasized because it increases the baseline probability of pregnancy and limits generalizability to the anoestrous population.

Females ranged from 14 to 96 months of age. At the time of progesterone device insertion (Day 0), heifers (14–24 months) were required to reach a minimum body weight of approximately 150 kg, and multiparous cows (≥ 36 months), including lactating cows, were evaluated for uterine involution through rectal palpation (absence of uterine contents and evidence of uterine regression). It is acknowledged that this minimum weight is at the lower end of that usually recommended for FTAI in Nelore heifers, which is considered when interpreting the results.

Body condition score (BCS) was assessed visually on Day 0 by trained veterinarians using a 1–5 scale (1 = emaciated and 5 = obese) (Ayres et al. 2009). For statistical analysis, females were classified into three categories: low BCS (< 2.5), moderate BCS (2.5–3.5), and high BCS (> 3.5). Scoring was based on fat deposition and muscle coverage at key anatomical points, including the ribs, lumbar region, hips, and tailhead. These three categories (low, moderate, high) are used consistently throughout the manuscript, including the tables.

Only the first FTAI performed per animal during the breeding season was considered, excluding repeated inseminations within the same individual.

The FTAI protocol was conducted as follows: on Day 0 (D0), all animals received an intravaginal progesterone device containing 1.9 g of P4 (CIDR^®^, Zoetis), which could be used for the 1st, 2nd, 3rd, or 4th time, along with an intramuscular injection of 2 mg estradiol benzoate (Gonadiol^®^, Zoetis). On Day 7 (D7), 12.5 mg dinoprost tromethamine (Lutalyse^®^, Zoetis) was administered intramuscularly. On Days 8, 9, or 10 (D8–D10), the progesterone device was removed, and animals received 0.6 mg estradiol cypionate (E.C.P.^®^, Zoetis) and 200 or 300 IU equine chorionic gonadotropin (eCG; Novormon^®^, Zoetis). Fixed-time artificial insemination was performed 48 h after device removal (Crepaldi 2009).

Pregnancy diagnosis was performed 30 days after insemination by transrectal ultrasonography (Mindray DP-10Vet, 7.5 MHz linear transducer) and/or rectal palpation, based on the detection of uterine changes indicative of pregnancy. Females were classified as non-pregnant (0) or pregnant (1).

### Statistical analysis

Pregnancy per FTAI (pregnant = 1; non-pregnant = 0) was the outcome. The associations of female category (heifer, multiparous), BCS category (low, < 2.5; moderate, 2.5–3.5; high, > 3.5), day of device removal (D8, D9, D10) and number of device uses (1st–4th) with pregnancy were estimated using multivariable logistic regression in which all factors were modelled jointly, so that each estimate was adjusted for the remaining factors. To account for the clustering of females within farms, farm was included as a random effect (mixed-effects logistic regression); estimates were confirmed using generalized estimating equations with an exchangeable working correlation structure and farm as the clustering unit, which provide cluster-robust standard errors. Category × factor interactions (category × BCS, category × removal day, category × device use) were tested; because interactions involving female category were detected, the analyses were stratified by category (heifers and multiparous cows), and an overall model adjusted for category was also fitted. Associations are reported as odds ratios (OR) with 95% confidence intervals (CI). Pairwise contrasts within each factor were derived from the fitted models, and their p-values were adjusted for multiple comparisons using the Holm procedure; exact adjusted p-values are reported. For device use, the difference at the fourth use was additionally assessed with a single pre-specified contrast (fourth use versus first-to-third uses). Statistical significance was set at *p* < 0.05. Analyses were performed in SAS 9.4 (PROC GLIMMIX with farm as a random effect; PROC GENMOD with a repeated statement for the generalized estimating equations). As the study is retrospective and observational, results are interpreted as associations rather than as causal effects.

## Results

The overall pregnancy rate was 51.5% and did not differ between heifers (50.9%) and multiparous cows (51.8%; *p* > 0.05; Table 1).

Body condition score was associated with pregnancy in multiparous cows and in the overall population, but not in heifers. In multiparous cows, the odds of pregnancy were higher for high- than for moderate-BCS cows (OR 1.90; 95% CI 1.24–2.90; *p* = 0.003) and than for low-BCS cows (OR 2.40; 1.56–3.71; *p* < 0.001), and higher for moderate- than for low-BCS cows (OR 1.27; 1.11–1.45; *p* = 0.001). The same pattern was observed in the overall population (high vs. low OR 1.94; 1.26–2.99; *p* = 0.005; moderate vs. low OR 1.28; 1.12–1.45; *p* < 0.001). In heifers, no pairwise contrast reached significance (e.g., moderate vs. low OR 1.28; 0.99–1.65; *p* = 0.17; Table 1).

**Table 1:** Pregnancy rate (%) and odds ratios (OR) for pregnancy across body condition score categories in Nelore females submitted to FTAI

Body condition score	Heifers (*n* = 10,079)	Multiparous (*n* = 21,279)	Overall (*n* = 31,358)
High (> 3.5)	53.6 (45/84)	68.3 (69/101)	61.6 (114/185)
Moderate (2.5–3.5)	51.0 (4,955/9,716)	52.5 (9,701/18,461)	52.0 (14,656/28,177)
Low (< 2.5)	46.6 (130/279)	46.3 (1,257/2,717)	46.3 (1,387/2,996)
*High vs. Moderate*	1.07 (0.70–1.64); *p* = 0.746 ns	1.90 (1.24–2.90); *p* = 0.003 *	1.52 (1.01–2.28); *p* = 0.043 *
*High vs. Low*	1.37 (0.84–2.24); *p* = 0.403 ns	2.40 (1.56–3.71); *p* < 0.001 *	1.94 (1.26–2.99); *p* = 0.005 *
*Moderate vs. Low*	1.28 (0.99–1.65); *p* = 0.166 ns	1.27 (1.11–1.45); *p* = 0.001 *	1.28 (1.12–1.45); *p* < 0.001 *

Pregnancy rate is shown as % (pregnant/inseminated). Contrast cells show OR (95% CI) and the Holm-adjusted p-value from multivariable logistic regression accounting for farm clustering; * *p* < 0.05, ns = not significant

After adjustment for the remaining factors and for farm clustering, the day of progesterone device removal (D8–D10) was not associated with pregnancy in heifers, multiparous cows, or the overall population; no pairwise contrast was significant (all *p* > 0.6; Table 2). Although pregnancy rates were numerically slightly higher with later removal (overall 49.9%, 51.7% and 53.3% for D8, D9 and D10, respectively), these differences did not persist in the multivariable, clustered analysis.

**Table 2:** Pregnancy rate (%) and odds ratios (OR) for pregnancy across days of progesterone device removal in Nelore females submitted to FTAI

Day of device removal	Heifers (*n* = 10,079)	Multiparous (*n* = 21,279)	Overall (*n* = 31,358)
D8	46.8 (570/1,217)	51.0 (1,609/3,152)	49.9 (2,179/4,369)
D9	51.3 (4,316/8,420)	51.9 (8,815/16,979)	51.7 (13,131/25,399)
D10	55.2 (244/442)	52.5 (603/1,148)	53.3 (847/1,590)
*D8 vs. D9*	0.85 (0.63–1.14); *p* = 0.857 ns	0.93 (0.79–1.11); *p* = 0.665 ns	0.92 (0.80–1.05); *p* = 0.639 ns
*D8 vs. D10*	0.89 (0.38–2.09); *p* = 1.000 ns	0.76 (0.49–1.18); *p* = 0.665 ns	0.79 (0.55–1.14); *p* = 0.639 ns
*D9 vs. D10*	1.05 (0.54–2.01); *p* = 1.000 ns	0.82 (0.55–1.21); *p* = 0.665 ns	0.86 (0.63–1.18); *p* = 0.639 ns

Pregnancy did not differ significantly among the first, second and third device uses in any category, and no pairwise contrast among individual uses remained significant after correction for multiple comparisons (Table 3). In multiparous cows, a pre-specified contrast comparing the fourth use with the first-to-third uses indicated lower odds of pregnancy at the fourth use (OR 0.80; 95% CI 0.66–0.98; *p* = 0.028); the corresponding pregnancy rate at the fourth use was 45.0%, versus 51.6–53.7% at earlier uses. No such association was observed in heifers.

**Table 3:** Pregnancy rate (%) and odds ratios (OR) for pregnancy across progesterone device uses in Nelore cows submitted to FTAI

Device use	Heifers (*n* = 10,079)	Multiparous (*n* = 21,279)	Overall (*n* = 31,358)
1st	55.3 (745/1,348)	51.6 (4,996/9,686)	52.0 (5,741/11,034)
2nd	51.0 (1,444/2,830)	53.0 (2,698/5,091)	52.3 (4,142/7,921)
3rd	48.9 (1,135/2,321)	53.7 (2,515/4,685)	52.1 (3,650/7,006)
4th	50.4 (1,806/3,580)	45.0 (818/1,817)	48.6 (2,624/5,397)
*1 vs. 2*	1.03 (0.87–1.22); *p* = 1.000 ns	0.82 (0.62–1.09); *p* = 0.708 ns	0.90 (0.72–1.12); *p* = 1.000 ns
*1 vs. 3*	1.11 (0.89–1.37); *p* = 1.000 ns	0.88 (0.73–1.06); *p* = 0.708 ns	0.93 (0.80–1.08); *p* = 1.000 ns
*1 vs. 4*	1.09 (0.92–1.29); *p* = 1.000 ns	1.13 (0.90–1.42); *p* = 0.708 ns	1.04 (0.86–1.25); *p* = 1.000 ns
*2 vs. 3*	1.07 (0.90–1.27); *p* = 1.000 ns	1.07 (0.75–1.53); *p* = 0.708 ns	1.03 (0.84–1.27); *p* = 1.000 ns
*2 vs. 4*	1.06 (0.89–1.26); *p* = 1.000 ns	1.38 (1.03–1.85); *p* = 0.190 ns	1.15 (0.94–1.41); *p* = 1.000 ns
*3 vs. 4*	0.98 (0.79–1.22); *p* = 1.000 ns	1.28 (1.00–1.65); *p* = 0.260 ns	1.12 (0.95–1.32); *p* = 1.000 ns

A pre-specified contrast (fourth use vs. first-to-third uses) in multiparous cows gave OR 0.80 (0.66–0.98); *p* = 0.028

## Discussion

The overall pregnancy rate (51.5%) was consistent with values commonly reported for FTAI programmes in Bos indicus beef cattle (around 50%) and did not differ between heifers and multiparous cows, in agreement with previous Brazilian reports (Carvalho et al. 2019; Grillo et al. 2015) and with national data from the GERAR group (about 51.6% across ~ 1.4 million inseminations). Obtaining a comparable estimate from a large, multi-farm dataset reinforces the robustness of this figure; nevertheless, the absence of a category difference should be interpreted in light of the enrolment of cyclic females with adequate body weight, discussed below.

Body condition score was the factor most consistently associated with pregnancy, and this association was restricted to multiparous cows. Because only cyclic females were enrolled, the association is unlikely to reflect a simple failure to resume cyclicity; rather, within an already-cyclic population, lower BCS in multiparous cows, many of which were lactating, probably reflects a more negative energy balance that compromises follicular and oocyte quality, the peri-ovulatory endocrine environment, and early embryo survival, thereby reducing the probability of pregnancy (Filho et al. 2009; Ferreira et al. 2013; Ayres et al. 2014). This interpretation reconciles the enrolment of cyclic females with the observed BCS association and is consistent with the recommendation to maintain BCS at or above 3.0 to optimise fertility, and with the association between adequate BCS and reduced embryonic loss in Bos indicus females (Ayres et al. 2014; Sá Filho et al. 2013).

In heifers, BCS was not associated with pregnancy. This is consistent with the selection criteria applied, since only cyclic heifers that had reached the target body weight were inseminated; under these conditions, attainment of puberty and adequate body weight, rather than BCS per se, are considered the primary determinants of fertility (Sá Filho et al. 2013; Baruselli et al. 2017). It should be noted, however, that some heifers were enrolled at a relatively low body weight, which may limit the generalisability of this particular finding. Comparable outcomes have likewise been reported for heifers submitted to progestin-based FTAI protocols in Bos indicus and crossbred genotypes, in which body weight and cyclicity, rather than body condition score, discriminated fertility (Bonato et al. 2015).

The day of progesterone device removal (D8–D10) was not associated with pregnancy once all factors were modelled jointly and farm clustering was taken into account. The numerically higher rates observed with later removal did not persist after adjustment, indicating that they were largely attributable to differences between farms and to the uneven distribution of animals across removal days rather than to the length of progesterone exposure itself. This null association is in line with studies that reported no effect of removal timing on ovulation or pregnancy (Santos 2016, 2018) and tempers reports of benefits from longer exposure (Roman 2018). Importantly, extending progesterone exposure is not universally beneficial: excessively prolonged dominance of the ovulatory follicle can generate persistent follicles with reduced oocyte competence and lower fertility (Austin et al. 1999; Revah and Butler 1996) so longer protocols should not be assumed to improve outcomes. Within the 8–10-day window studied, removal day therefore appears to offer operational flexibility without a measurable association with pregnancy.

Similarly, reuse of progesterone devices up to the third time was not associated with pregnancy in any category, consistent with reports of no detrimental effect of moderate reuse (Rocha et al. 2007; Meneghetti et al. 2009; Medalha et al. 2015; Santos et al. 2020; Batata et al. 2021). In multiparous cows, however, a pre-specified comparison indicated that the fourth use was associated with lower odds of pregnancy than earlier uses; because the individual pairwise contrasts among uses did not survive correction for multiple comparisons, this signal should be regarded as suggestive rather than definitive. A plausible mechanism is that extensively reused devices release insufficient progesterone to adequately regulate luteinising-hormone secretion and follicular dynamics (Valentim 2004; Sá Filho et al. 2011); however, as circulating progesterone was not measured, this remains speculative. From a practical standpoint, device reuse up to the third time appears to be a viable cost-saving strategy, whereas the fourth use in multiparous cows should be adopted with caution.

Some limitations should be considered when interpreting these findings. First, this is a retrospective, observational analysis; the reported associations do not establish causation, and residual confounding cannot be excluded despite the inclusion of farm as a random effect. Second, enrolment was restricted to cyclic females without uterine infection and, for heifers, to animals meeting a minimum weight; this selection increases the baseline probability of pregnancy and limits external validity to comparable, well-managed populations rather than to the general herd, which also includes anoestrous and lighter females. Third, some potentially relevant variables were not available in the dataset, including lactation status of multiparous cows, semen and sire fertility, inseminator, and the specific method of pregnancy diagnosis, which may contribute to residual between- and within-farm variation. Finally, BCS was assessed visually and circulating progesterone was not measured. These limitations should be addressed in prospective, adequately controlled studies.

## Conclusion

In cyclic Nelore females subjected to FTAI under commercial field conditions, body condition score in multiparous cows was the main factor associated with pregnancy, with lower BCS associated with lower pregnancy odds; no such association was observed in heifers, likely reflecting their selection for adequate weight and cyclicity. Within the 8–10-day window evaluated, the day of progesterone device removal was not associated with pregnancy. Progesterone devices could be reused up to the third time without a detectable reduction in pregnancy, whereas the fourth use in multiparous cows was associated with lower pregnancy odds and should be used with caution. Interpreted as associations rather than causal effects, these findings identify nutritional management aimed at maintaining adequate BCS in multiparous cows as the most actionable lever for improving reproductive efficiency in these systems, and support device reuse up to three times as a cost-effective practice.

## Data Availability

The datasets generated during and/or analysed during the current study are not publicly available, but are available from the corresponding author on reasonable request.
